# Resveratrol Alleviating the Ovarian Function Under Oxidative Stress by Alternating Microbiota Related Tryptophan-Kynurenine Pathway

**DOI:** 10.3389/fimmu.2022.911381

**Published:** 2022-07-13

**Authors:** Jianping Wang, Ru Jia, Pietro Celi, Yong Zhuo, Xuemei Ding, Qiufeng Zeng, Shiping Bai, Shengyu Xu, Huadong Yin, Li Lv, Keying Zhang

**Affiliations:** ^1^ Animal Disease-Resistance Nutrition, Ministry of Education, Ministry of Agriculture and Rural Affairs, Key Laboratory of Sichuan Province, Animal Nutrition Institute, Sichuan Agricultural University, Chengdu, China; ^2^ Faculty of Veterinary and Agricultural Sciences, The University of Melbourne, Parkville, VIC, Australia

**Keywords:** follicle atresia, SIRT1-P53/FoxO1 pathway, oxidative stress, ovarian function, inflammation cytokines

## Abstract

Oxidative stress (OS) is a key factor regulating the systemic pathophysiological effects and one of the fundamental mechanisms associated with aging and fertility deterioration. Previous studies revealed that resveratrol (RV) exhibits a preventive effect against oxidative stress in the ovary. However, it remains unknown whether gut microbiota respond to resveratrol during an OS challenge. In Exp. 1, layers received intraperitoneal injection of tert-butyl hydroperoxide (tBHP) (0 or 800 μmol/kg BW) or received resveratrol diets (0 or 600 mg/kg) for 28 days. In Exp. 2, the role of intestinal microbiota on the effects of resveratrol on tBHP-induced oxidative stress was assessed through fecal microbiota transplantation (FMT). The OS challenge reduced the egg-laying rate and exhibited lower pre-hierarchical follicles and higher atretic follicles. Oral RV supplementation ameliorated the egg-laying rate reduction and gut microbiota dysbiosis. RV also reversed the tryptphan-kynurenine pathway, upregulated nuclear factor E2-related factor 2 (Nrf2) and silent information regulator 1(SIRT1) levels, and decreased the expression of forkhead box O1 (FoxO1) and P53. These findings indicated that the intestinal microbiota-related tryptophan-kynurenine pathway is involved in the resveratrol-induced amelioration of ovary oxidative stress induced by tBHP in the layer model, while SIRT1-P53/FoxO1 and Nrf2-ARE signaling pathway were involved in this process.

## Highlights

1. Resveratrol significantly alleviated tBHP-induced oxidative stress on the laying rate and follicle numbers.
2. Resveratrol activated the SIRT1/FoxO1 and Nrf2 pathways leading to enhanced antioxidant gene expression.
3. Intestinal microbiota sustained tryptophan metabolism through the kynurenine pathway plays a crucial role in ovary oxidative stress.

## Introduction

As the most important reproductive organ in females, the ovary plays a vital role in the maintenance of reproductive potential and endocrine stability. Recently, growing interest has been paid to the role of oxidative stress (OS) in female fertility (1, 2). OS is the resultant damage due to redox imbalance and has been proven to be involved in the internal mechanism of aging (3–5), many environmental stressors (heat stress, pollutants, gamma radiation, overcrowding, etc.), and health disorders (6–9). Moreover, it is evident from the previous studies that OS also leads to follicular atresia and ovarian aging (10–12). Recent studies indicated that OS can affect nutrient metabolism by exerting detrimental influences on intestinal function, gut microbiota, and altering the body’s homeostasis in mammals and poultry (8, 13, 14). Moreover, OS was also found to be related to chronic inflammation by activating a variety of transcription factors, which leads to the differential expression of some genes involved in inflammatory pathways (15). However, the exact underlying molecular mechanisms in OS-induced fertility deterioration remain largely unknown.

Resveratrol (RV) is a widely available polyphenol and shows anti-aging and prevents ovarian aging effects (5, 16, 17). The anti-aging mechanisms of RV are associated with its antioxidant properties, as it can scavenge superoxide, hydroxyl radicals, and peroxynitrite (5, 18, 19). In addition to scavenging ROS, RV can activate silent information regulator 1 (SIRT1) to promote mitochondrial function (20). SIRT1 is the key metabolic sensor and mediates the response to oxidative stress resistance (21–23). SIRT1 was found to be ubiquitously expressed in animal ovaries and has a regulating role in oocyte maturation and ovarian aging (23–25). FoxO1 (forkhead box O1), a transcription factor downstream of SIRT1, is also involved in the regulation of cell fate and combating oxidative stress (24, 26). Did the SIRT1-FoxO1 pathway involve the anti-oxidative stress function of resveratrol in the ovary? RV also was found to activate the nuclear factor E2-related factor 2 (Nrf2)/antioxidant defense pathway (27). Nrf2 is an emerging regulator of cellular resistance to toxic xenobiotic substances and oxidants. Moreover, emerging evidence has indicated that the beneficial effects of RV were also closely related to gut microbiota (18, 28). Tryptophan catabolism was in relation to inflammation and immune response in oxidative stress and many other pathological disorders (29–31). However, the effects of oxidative stress on tryptophan metabolism and response to RV remain unknown.

Therefore, we hypothesized that resveratrol administration would modulate microbiota-related tryptophan metabolism and maintain ovarian function through the SIRT1-FoxO1 and Nrf2 signaling pathway in this study. Thus, since the avian ovary can be used as a model organism to study ovarian biology and the development of ovarian cancer, the aim of this study was to investigate the alleviating effect of resveratrol on ovary function under oxidative stress in a layer model.

## Materials and Methods

### Animals, Diets, and Design

**Figure 1:**
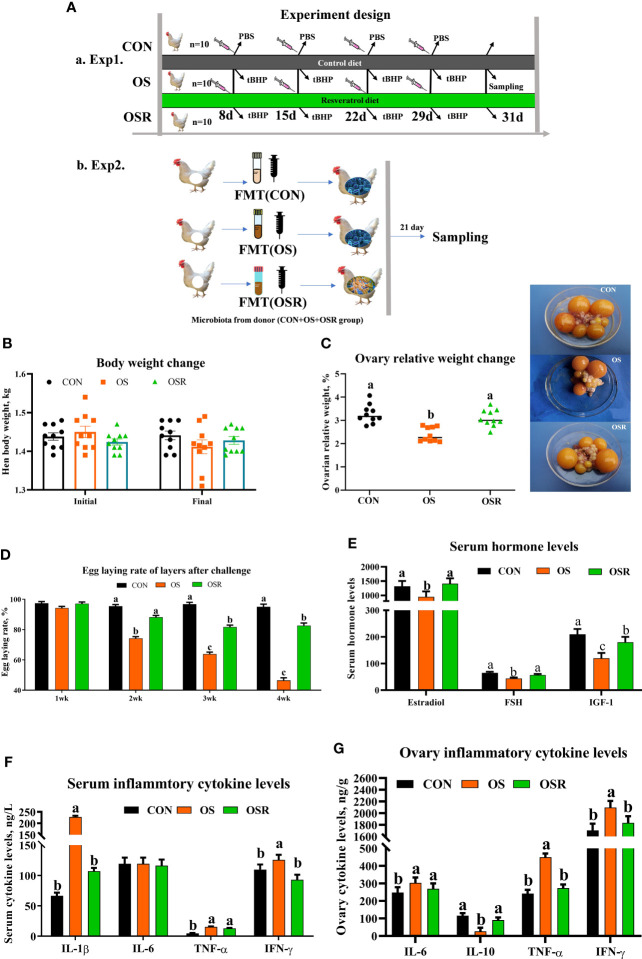
Dietary resveratrol supplementation alleviated the oxidative stress (by tBHP) induced depression in egg-laying rates, reproductive hormones, and cytokine levels. Data are means ± SEM represented by vertical bars or plot individual values ± SEM. **(A)** Schematic illustration of the experimental design. a, in experiment 1, layers were fed the same basal diet for 24 days and with the tBHP (OS) or PBS (CON) injection at 9 AM on d 8, 15, and 21. The OSR group diet was supplemented with 600 mg/kg resveratrol added in CON treatment. b, in experiment 2, the microbiota-depleted layers were fed CON diet and received FMT from donor mice (n= 10/group) that were fed resveratrol. **(B)** Body weight change. **(C)** Ovary index. **(D)** Egg-laying rate after challenge. **(F)** Serum hormone levels. **(G)** Serum cytokine levels. **(D)** Ovary cytokine levels. CON, control; OS, oxidative stress (injection of 800 μmol/kg BW of tBHP); OSR, CON + 600 mg/kg resveratrol; FSH, follicle-stimulating hormone; IGF-1; insulin-like growth factor-1, IL-1β = interleukin-1 β; IL-6, interleukin-1, IL-10, interleukin-10; TNF-α, tumor necrosis factor alpha; IFN-γ, Interferon-γ. Statistical significance was evaluated by Tukey’s Test. *p* < 0.05.

The Animal Care and Use Committee of Sichuan Agricultural University (SICAU-2020-041) approved all the protocols used in the present study. Thirty Lohmann laying hens at 25 wks of age (BW = 1.44 ± 0.10 kg) raised on the same farm (Teaching and Research Center of Sichuan Agricultural University, Ya’an, China) were chosen according to their laying rate and body weight and randomly assigned to three treatments (n = 10). All birds were fed a basal diet and received an intraperitoneal injection of either PBS (CON) or tert-butyl hydroperoxide (tBHP; 800 μmol/kg BW; according to 8) at 9 am on the 8^th^, 15^th^, 22^nd^, and 29th day of the 31-d experiment (OS), and injection of tBHP and dietary supplementation of 600 mg/kg resveratrol (OSR). The experiment protocol is presented in **Figure 1Aa**. All hens were kept under controlled environmental conditions with the maintained temperature at 22 ± 2°C with relative humidity at 45%-60%). The lighting cycle was kept at 16 h/d; 5:00 a.m. to 9:00 p.m. for light. Layers were subjected to the same amount of feed (110 g/kg/d/hen; **Supplementary Table S1**) and had free access to water.

### Fecal Transfer Experiment

Twenty microbiota-depleted Lohmann layers (1.41 ± 0.12 kg, 28 wk of age) were chosen for the fecal transfer experiment and the procedure is depicted in **Figure 1Ab**. Recipient layers (n = 10) were treated with antibiotics (penicillin 1 g/L and streptomycin1 g/L) for 7 consecutive days in order to deplete endogenous gut microbiota before the fecal transfer, as previously described (28, 32). Then, the microbiota-depleted layer that was originally fed the control diet received FMT from donor layers (n = 10) that were raised with CON [FMT (OS), OSR (OS + resveratrol]. 16S rRNA gene sequencing was used to validate the depletion of microbiota. For FMT, approximately 500 g of fresh fecal excreta were extracted *via* donor breeder followed by resuspension in 2.5 L of sterile PBS (0.1 M, pH7.2) under anaerobic conditions. Then, the supernatant was collected and FMT was performed by a single oral administration of 2 mL of suspension (1.4×10^12^ CFU/mL) according to the method described previously (28, 33).

### Sample Collection and Measurements

After 12 h of fasting on the final day, blood samples (n = 10) were obtained from the wing vein and blood samples were centrifugation at 1500×g at 4°C for 30 min to separate the serum. The birds taken for blood samples were then sacrificed and the ovarian tissue was immediately taken for further ovarian follicle counts (H&E) and cell apoptosis (TUNEL) assay. The cecum mucosa and its contents were immediately collected and stored at -80°C until further analysis.

### Reproduction Performance, Blood Hormone, and Tryptophan Assay

The hen’s (n =10) body weight was weighed on the initial and final day of the experiment and the egg-laying numbers were recorded every day. The serum concentration of 17β-estradiol (E2, CHEB0528, ELISAGenie, Dublin, Ireland), follicle-stimulating hormone (FSH, CHFI00020, ELISAGenie), melatonin (RE54021, IBL, Germany), insulin-like growth factor-1 (IGF-1, CHFI00088, ELISAGenie), and serotonin (ab133053, Abcam, UK) were measured using ELISA kits followed by the manufacturer’s instructions.

### Serum and Ovarian Inflammatory Cytokine Expression

The serum (n =10) and ovary (n = 10) samples were used to evaluate the concentration of tumor necrosis factor-α (TNF-α), interleukin-1β (IL-1β), IL-6, IL-10, and interferon-γ (IFN-γ) by ELISA kits obtained from Nanjing Jiancheng Bioengineering Institute (Nanjing, China) according to the manufacturer’s instructions.

### Tissue Antioxidant Capacity and Tryptophan Metabolism-Related Activities

**Antioxidant Capacity**

Serum and ovary tissue (n = 10) were used to determine the antioxidant capacity, including activities of glutathione (GSH) peroxidase (GSH-Px), GSH s-transferase (GSH-ST), superoxide dismutase (SOD), and the contents of GSH, total antioxidant capacity (T-AOC), protein carbonyl (PC), and malondialdehyde (MDA) which were measured by commercial kits (Nanjing Jiancheng Bioengineering Institute, Nanjing, China) according to the manufacturer’s manual without any modification.

#### Serum Tryptophan and Kynurenine Level

Reverse-phase high-pressure liquid chromatography was applied to analyze the free tryptophan and kynurenine concentrations in serum (n = 10). Kynurenine contents were determined by UV-absorption at 360 nm wavelength, while tryptophan was measured at an excitation wavelength of 285 nm and an emission wavelength of 365 nm by a fluorescence detector (34).

#### Indoleamine 2,3-Dioxygenase (IDO)1 Activity

Jejunum tissues (n = 10) were homogenized in a Potter-Elvehjem homogenizer with a Teflon pestle and centrifuged at 12,000 × g for 30 min at 0°C. The resulting supernatants were used to analyze indoleamine 2,3-dioxygenase (IDO)1 activity followed by the method described previously (35).

### Histology and Follicle Counts

Ovarian tissue samples (n = 10) were placed into 4% paraformaldehyde (pH = 7.2) for 24 h, embedded in paraffin, serially sectioned into 5 μm thickness using a microtome, and stained with hematoxylin. Ovarian follicles were categorized into different stages according to a previous study (8). The follicles were divided into three stages: primordial follicle (consisting of an oocyte surrounded by a single layer of flattened five to eight somatic cells), primary follicles (consisting of an oocyte surrounded by one layer consisting of an enlarged cell or a whole layer of cuboidal pre-granulosa cells), and prehierarchical follicles, which included SYFs (small yellow follicle, 6-8 mm), LWFs (large white follicles, 4-6 mm), and SWFs (small white follicles, 2-4 mm diameters) (36). Atretic follicles were defined as previously described (8, 37).

### TUNEL Assay

Ovarian cell apoptosis rate (n = 10) was assessed by TUNEL assay using an apoptosis detection kit (Roche, Basel, Switzerland). Paraffin sections of ovaries were deparaffinized and washed twice with PBS and then stained with a TUNEL reaction mixture. After washing twice with PBS, the sections were counter-stained with DAPI. Blue color with a white background indicated negative expression, while light yellow or brown yellow referred to the positive cell (apoptosis).

### Ovary Function-Related mRNA Expression by Real-Time PCR

The total RNA from ovarian tissues (n = 10) was extracted with TRIzol Reagent (Life Technologies, Carlsbad, CA, USA) using RQ1 RNase-free DNase to remove genomic DNA. The primer information for all the genes [*Bax, Bcl-2(B-cell lymphoma-2)*, *caspase 3, caspase 9, SIRT1, FoxO*, and *P53*] is listed in **Supplementary Table S2**. Gene expression was determined by quantitative real-time PCR by ABI 7900 Real-Time PCR system (ABI Biotechnology, Eldersburg, MD, USA). Each sample was assayed in triplicate and relative quantitative analysis of the results was normalized to β–actin by using the 2*^-^* ^ΔΔCT^ method.

### Western Blotting Assay

Total protein expression in ovary tissues (n =10) was analyzed by Western blotting as previously described (28). Primary antibodies against FoxO1 (#9454S), P53 (#9282S), SIRT1 (#2310S), and β-actin (#4970S) were obtained from Cell Signaling Technology. Blots were quantified with Image J software (National Institutes of Health, Bethesda, MD, USA).

### 2.11 Gut Microbiota and Short-Chain Fatty Acids (SCFA) Analysis

To investigate the changes in the cecal microbiota, microbial genomic DNA was extracted from each sample (n = 10) using QIAamp Fast DNA Stool Mini Kit (Qiagen, Germany) according to the manufacturer’s instructions. An enzyme standard instrument (Multiskan™ GO) was used to detect the concentration of the extracted DNA. The Illumina library construction strategy was used to build a 16S library, quantified using Qubit 3.0. Out-machine double-ended data (raw reads) of samples were obtained using the Illumina HiSeq/MiniSeq sequencing platform. The sequencing and bioinformatics analysis were performed by Novogene Bioinformatics Technology Co. (Tianjin, China).

The short-chain fatty acids were measured on an Agilent 6890 gas chromatograph (Agilent Technologies, Santa Clara, CA, USA) according to the protocol previously described (38).

### Metabolic Profiling Analysis

The serum samples (n = 10) were used for the identification and quantification of specific endogenous metabolites among treatments. GC-MS-based metabolomics was performed by Novogene Bioinformatics Technology Co. (Tianjin, China) according to the previous study (8). Differences were indicated when the P-value was < 0.05, VIP (Variable Importance in the Projection) > 1, and only fold changes > 1.5 were considered.

### Statistical Analysis

One-way ANOVA using the GLM procedure of SAS 9.2 (SAS Institute, Cary, NC, USA) and GraphPad Prism (GraphPad Inc., La Jolla, CA, USA) was used to analyze the data. The results are presented as mean and SEM. Contrasts between treatments were evaluated by Tukey’s range test at a significance level of 0.05.

## Results

### Resveratrol Alleviated the Oxidative Stress (Caused by tBHP) Induced Depression in the Egg-Laying Rate, Reproductive Hormones, and Cytokine Levels

A reduction in the egg-laying rate, ovarian indices, serum estradiol, FSH, and IGF-1 concentration was observed in the OS group compared to the CON group (**Figures 1C–E**; *P* < 0.05); moreover, layers from the OS group presented a higher proinflammatory cytokine concentration (IL-1β, IL-6, TNF-α, and IFN-γ) in both serum (except IL-6) and ovary when compared to CON one (**Figures 1F, G**; *P* < 0.05). Dietary resveratrol supplementation was able to alleviate the OS-induced depression in the egg-laying rate, ovarian indices, and serum hormone levels, while it decreased the serum (IL-1β and IFN-γ) and ovarian (IL-1β, IL-6, TNF-α, and IFN-γ) proinflammatory cytokine levels. No differences in body weight were noted among the three groups (**Figure 1B**; *P* > 0.05).

### Resveratrol Alleviated Antioxidant Capacities and Changed Tryptophan Metabolism in the OS Model

**Figure 2:**
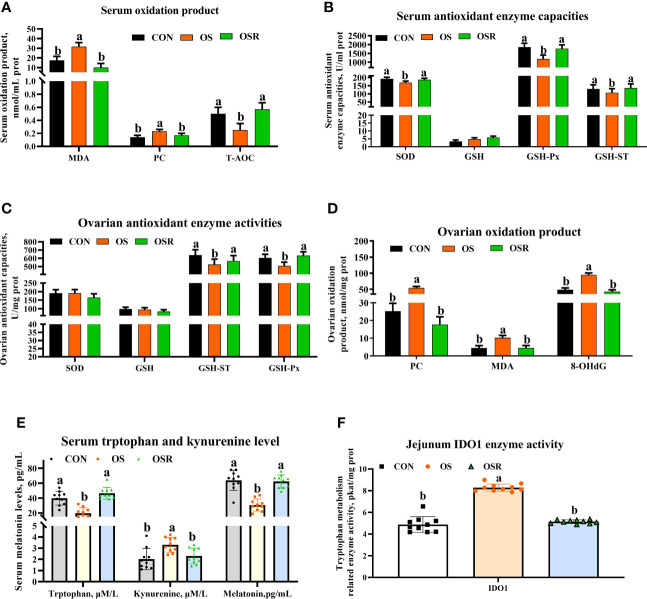
Dietary resveratrol supplementation alleviated the oxidative stress (by tBHP) induced depression in activities of antioxidant enzyme and tryptophan metabolism related enzyme levels (Exp. 1). **(A)** Serum oxidation product levels. **(B)** Serum antioxidant enzyme capacities. **(C)** Ovarian antioxidant enzyme activities. **(D)** Ovarian oxidation product levels. **(E)** Serum melatonin tryptophan levels. **(F)** Intestinal tryptophan metabolism related enzyme activities. CON, control; OS, oxidative stress (injection of 800 μmol/kg BW of tBHP); OSR, CON + 600 mg/kg resveratrol; MDA, malondialdehyde; PC, protein carbonyl; 8-OHdG, 8-hydroxy-2’ -deoxyguanosine; T-AOC, total antioxidant capacity; SOD, superoxide dismutase; GSH, glutathione; GSH-ST, glutathione S-transferase; GSH-Px, glutathione peroxidase; IDO1, indoleamine 2,3-dioxygenase (IDO) 1. Statistical significance was evaluated by Tukey’s Test, *p* < 0.05.

Compared to the CON group, layers challenged with tBHP had lower antioxidant enzyme activities (SOD [only in serum], GSH-Px, GSH-ST) and higher peroxidation products (PC, MDA, and 8-OHDG [only in serum]) in serum and ovary (**Figures 2A–D**; *P* < 0.05), suggesting that the tBHP challenge induced oxidative stress in the ovary. Serum tryptophan and melatonin levels were also decreased and kynurenine increased in the OS group compared to the CON one, while serum tryptophan catabolism-related enzyme IDO1 activity increased (**Figures 2E, F**; *P* < 0.05). Dietary resveratrol supplementation mitigated the impact of oxidative stress-induced depression on antioxidant capacity (higher SOD, GSH-Px, GSH-ST in serum and ovary, lower MDA, PC, and 8-OHDG levels in ovary), while it also increased tryptophan and melatonin levels, but decreased kynurenine levels in serum, and changed tryptophan metabolism (lower IDO1 activity) (*P* < 0.05).

### Resveratrol Sustained Ovarian Function in the OS Model

**Figure 3:**
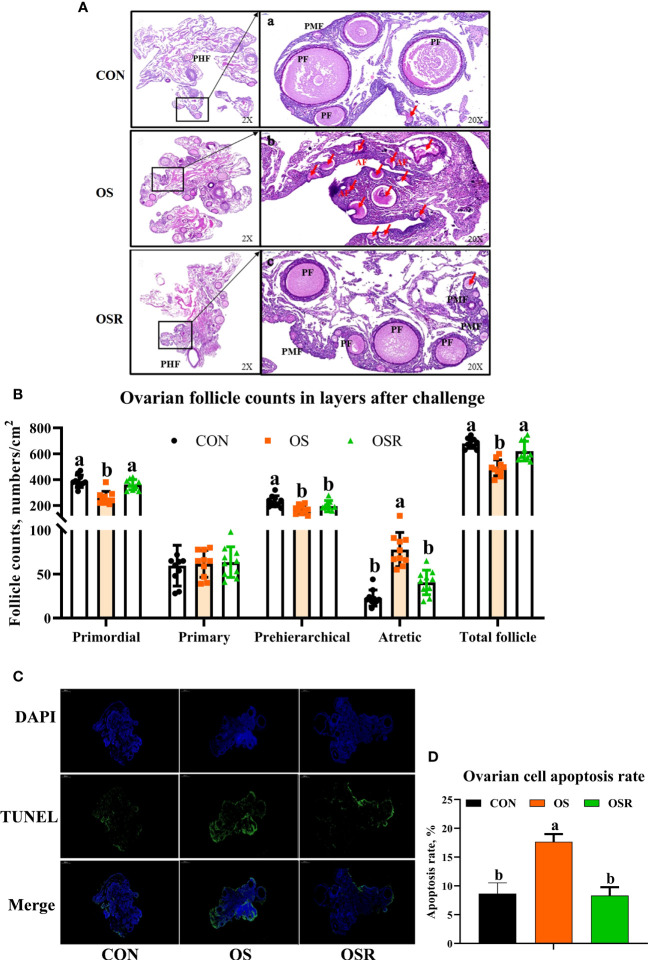
Dietary resveratrol supplementation alleviated the oxidative stress (by tBHP) induced depression in follicle counts and cell apoptosis (Exp. 1). **(A)** Ovarian histology of layers after 24 days of treatment. **(B)** Follicle numbers at each developmental stage (PMF= primordial follicle; PF = primary follicle; PHF = prehierarchical follicle; AF = atretic follicle). **(C)** TUNEL analysis of cell apoptosis in the ovary. **(D)** The immunofluorescence results of TUNEL with the green color representing the positive cells. Data are plot individual values (n = 10). CON, control; OS, oxidative stress (injection of 800 μmol/kg BW of tBHP); OSR, CON + 600 mg/kg resveratrol. Statistical significance was evaluated by Tukey’s Test, *p* < 0.05.

As presented in **Figures 3A, B**, the layers challenged with tBHP had lower numbers of follicles at each developmental stage (primordial, prehierarchical, total follicle) with the exception of primary follicles (*P* < 0.05), and had higher atretic follicle numbers and ovary apoptosis rates than in the CON ones (**Figures 3C, D**; *P* < 0.05). The layers from the OSR group had higher numbers of primordial, prehierarchical, and total follicles compared to OS treatment (*P* < 0.05), while dietary resveratrol supplementation mitigated the OS-induced increase in atretic follicle numbers and ovary apoptosis rates.

### Resveratrol Improved Ovarian Function by Inhibiting Apoptosis and Reducing Oxidative Stress in the OS Model

**Figure 4:**
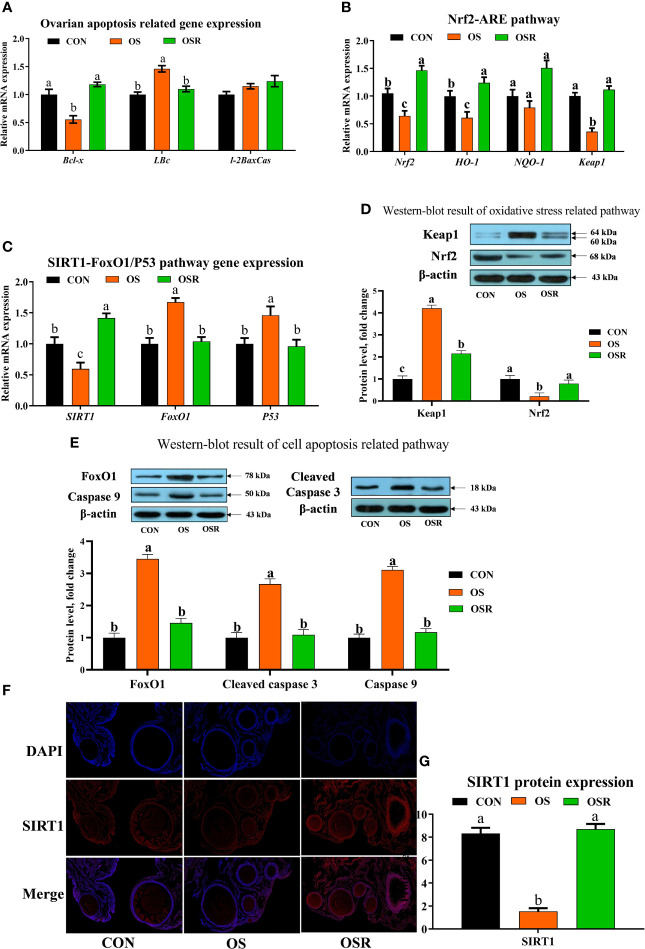
Dietary resveratrol supplementation alleviated the oxidative stress (induced by tBHP) induced depression ovarian oxidative stress and apoptosis-related pathway (Exp. 1). **(A)** Ovarian apoptosis-related gene expression. **(B)** Oxidative stress-related pathway (Nrf2-ARE) gene expression. **(C)** SIRT1-FoxO1/P53 gene expression. **(D)** Western-blot result of cell apoptosis-related pathway. **(F-G)** The immunofluorescence results of SIRT1 protein. Data are plot individual values (n = 10). CON, control; OS, oxidative stress (injection of 800 μmol/kg BW of tBHP); OSR, CON + 600 mg/kg resveratrol; SIRT1, silent information regulator 1; FoxO1, forkhead Box O1; Nrf2, nuclear factor E2-related factor 2. Statistical significance was evaluated by Tukey’s Test, *p* < 0.05.

As shown in **Figure 4**, mRNA proapoptotic factors (*Bax, FoxO1, P53*) and protein (*caspase 9, cleaved-caspase 3, FoxO1*) expression were upregulated in the ovary by OS, while the mRNA abundance of anti-apoptotic genes (*Bcl-2*, *SIRT1*) and antioxidant capacity-related genes (*Nrf2, HO-1, NQO-1, Keap1*) was lower in the OS group compared to the CON group (**Figures 4A–E**; *P* < 0.05). The ovaries of layers fed resveratrol had lower proapoptotic factors mRNA (*Bax, FoxO1, P53*) and protein (caspase 9, cleaved-caspase 3, FoxO1) expression than those in the OS group. The immunofluorescence results of SIRT1 showed that OS decreased SIRT1 expression while resveratrol supplementation increased SIRT1 intensity compared with the OS group (**Figure 4F ,G**; *P* < 0.05).

### Resveratrol Modulated Cecal Short-Chain Fatty Acids and Maintained Gut Microbiota Eubiosis in the OS Model

**Figure 5:**
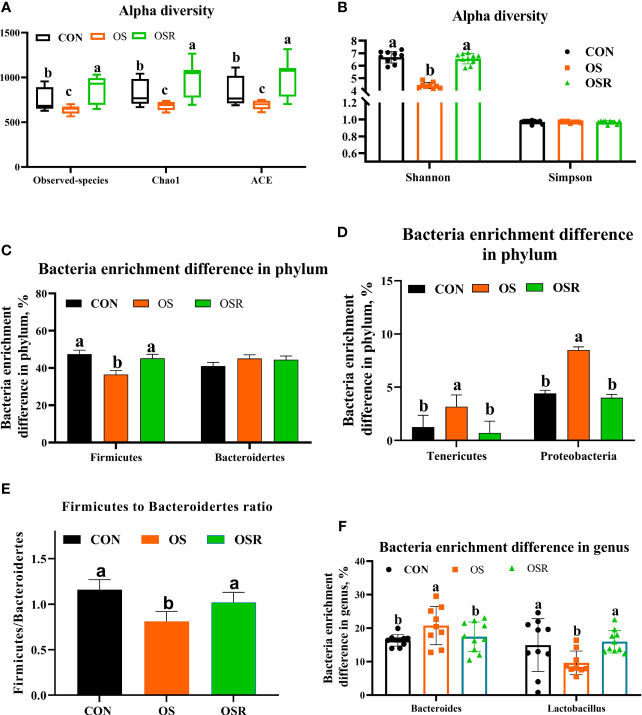
Dietary resveratrol supplementation alleviated the oxidative stress (by tBHP) induced reduction in cecal bacteria diversity and enrichment (Exp.1). **(A, B)** Alpha diversity of cecum microbiota, with observed species, Chao1, ACE**(A)**, and Shannon and Simpson index **(B)**. **(C-E)** Bacteria enrichment difference in phylum. **(F)** Bacteria enrichment difference in genus. Data are means ± SEM represented by vertical bars or plot individual values ± SEM. Statistical significance was evaluated by Tukey’s Test, p < 0.05.

**Figure 6:**
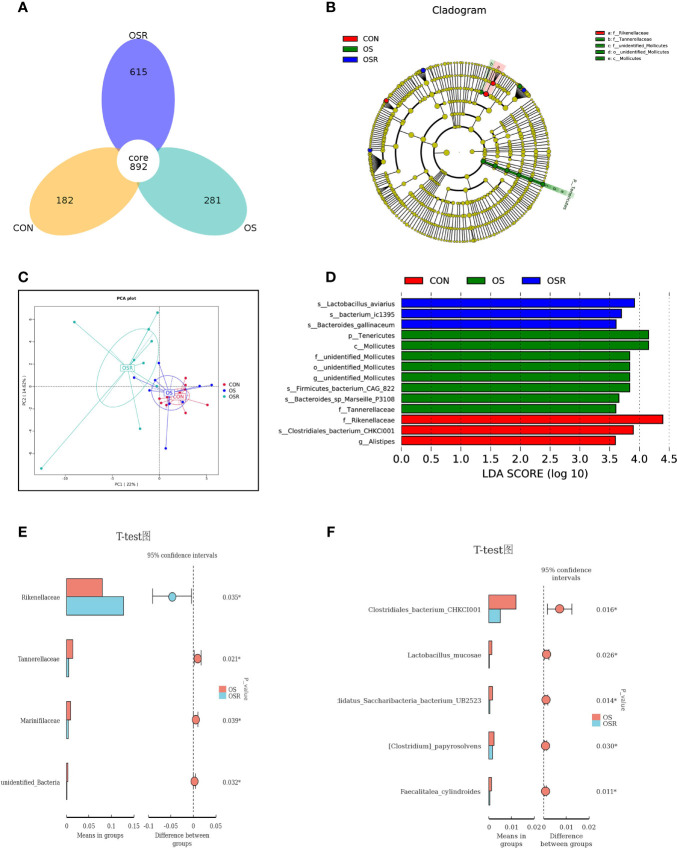
Dietary resveratrol supplementation modulated the oxidative stress (by tBHP) and induced a decrease in microbiota diversity (Exp.1). **(A)** Schematic illustration of the experimental design. **(A)** Vann diagram. **(C)** The principal coordinate analysis (PCoA) of the cecum microbiota based on unweighted UniFrac metric. **(B, D)** Linear discrimination analysis coupled with effect size (LEfse) identified the most differentially abundant taxa in the cecum with LDA significant threshold > 3 were shown. (Red) CON enrichment taxa; (Green) OS enrichment taxa; (Blue) OSR enrichment taxa. **(E,F)** Analysis of different species between groups at class **(E)** and species **(F)** levels. CON, same dosage of PBS; OS, 800 μmol/kg BW of tBHP. Statistical significance was evaluated by the Student’s T-test, * *p* < 0.05.

The layers fed resveratrol had higher cecum content concentrations of the main short-chain fatty acids (acetic, propionic, and butyric) and higher total SCFA than those observed in the CON and OS groups (**Supplementary Figure S1-A**; *P* < 0.05). As suggested by the decrease in the ACE, Chao1, and Shannon indices relative to the CON group, the tBHP challenge resulted in reduced microbial diversity (**Figures 5A–C**; **Figures S1B-D**; *P* < 0.05). Structural changes in intestinal microbiota, assessed by PCoA based on the unweighted UniFrac metric, indicated that layers in the three groups had obvious clustering (**Figure 6C**; *P* < 0.05). As analyzed by Adonis and LEfSe (log_10_LDA>3), the tBHP challenge significantly reduced the relative abundance of Firmicutes (phylum) and *Lactobacillus* (genus), while it led to an increase in the enrichment of *Proteobacteria* (Phylum), Tenericutes (phylum), and *Bacteroides* (genus) (**Supplementary Figures S1-B–D**; **Figures 5C–F**; **Figures 6A–F**; *P* < 0.05). The Firmicutes to *Bacteroidetes* ratio was also lower in the OS group compared with the CON group (**Figure 6**; *P* < 0.05). RV supplementation restored Simpson, ACE, Chao1, and Shannon indices reduced by tBHP (*P* < 0.05). Also, compared to the OS group, the OSR layers had higher enrichment in Firmicutes (phylum), *Rikenellaceae* (class), *Lactobacillus* (genus), and also OSR increased the Firmicutes to Bacteroidetes ratio, but it decreased the *Clostridiales_*bacterium*_*CHKC1001 (species), and *Lactobacillus_*mucosae (species) enrichment in the cecum.

### Resveratrol Changed Serum Metabolites of Layers During an OS Challenge

**Figure 7:**
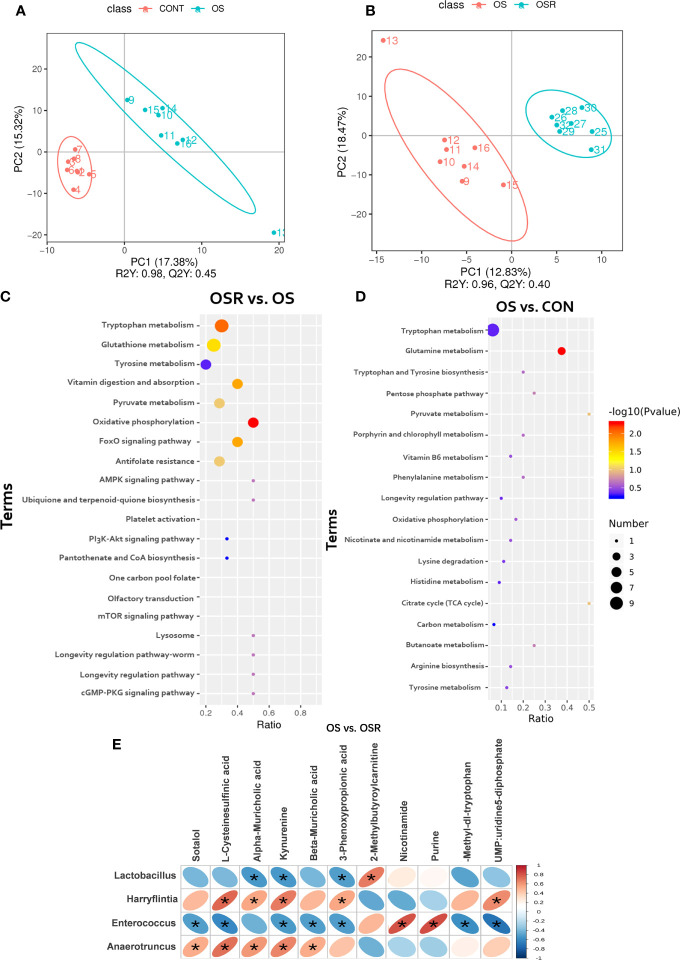
Dietary resveratrol supplementation alleviated the oxidative stress (by tBHP) induced changes in serum metabolites (Exp.1). **(A, B)** The principal component analysis (PCA) of the serum metabolites between OS vs. CON **(A)**, and between OSR vs. OS group **(B)**. **(C, D)** KEGG pathway enrichment of target metabolites between OS vs. CON **(C)**, and between OSR vs. OS group **(D)**. **(E)** Spearsman correlations between metabolites and cecum microbiota. Statistical significance was evaluated by the Student’s T-test, * *p* < 0.05.

**Figure 8:**
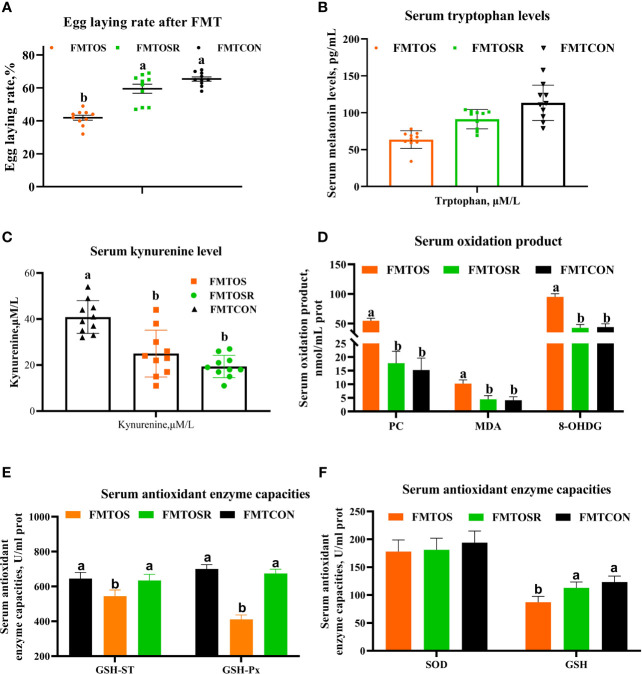
Egg-laying rate, serum melatonin, and antioxidant enzyme activities were alleviated by fecal microbiota transplantation from a resveratrol supplementation diet (Exp.2). Data are means ± SEM represented by vertical bars or plot individual values ± SEM. **(A)** Egg-laying rate. **(B,C)** Serum tryptophan and kynurenine levels. **(D-F)** Serum antioxidant capacities. CON = control, OS = oxidative stress (injection of 800 μmol/kg BW of tBHP), OSR = CON + 600 mg/kg resveratrol; FMTCON, FMTOS and FMTOSR indicated the microbiota-depleted layers received microbiota transplantations from donor layer treated CON diet, tBHP, and OSR, individually; MDA = malondialdehyde, PC = protein carbonyl, T-AOC = total antioxidant capacity, SOD = superoxide dismutase, GSH = glutathione, GSH-ST = glutathione S—transferase, GSH-Px = glutathione peroxidase. Statistical significance was evaluated by the Turkey’s T-test, *p* < 0.05.

In this study, a total of 486 metabolites were identified in the serum of the layers among CON, OS, and OSR groups; a notable metabolite alteration in OS and OSR was observed as compared to the CON or to the OS group, as shown by PLS-DA analysis (**Figures 7A, B**; **Supplementary Figures-S2-A–D**; *P* < 0.05). We observed that 19 metabolites were altered between the OS and CON groups, of which 12 of them were downregulated, and the remaining seven were upregulated (**Supplementary Table S1**; *P* < 0.05, VIP > 1). Also, 18 metabolites were different between the OSR and OS groups, of which 10 of them were upregulated and eight of them were downregulated (**Supplementary Table S2**, *P* < 0.05, VIP > 1). Most of those altered metabolites were classified into eight groups containing organic acids and derivates, amino acids, lipids and lipid-like molecules, nucleosides, aromatic heteropolycyclic compounds, aliphatic acyclic compounds, and aliphatic heteromonocyclic compounds. A high-quality KEGG metabolic pathways database was used to identify possible pathways relevant to the response of RV to oxidative stress (**Figures 7C, D**). As shown in **Figures 7C, D**, the concentration of differential metabolites in the corresponding metabolic pathways of Tryptophan metabolism, Purine metabolism, Tyrosine metabolism, FoxO signaling pathway, and Citrate cycle (TCA cycle) (*P* < 0.05). Correlations between most altered metabolites and microbiota response of RV to oxidative stress are shown in **Figure 8E** *via* Spearman’s correlation analysis. We observed that the bacteria genera, including *Lactobacillus*, *Harryflintia*, *Enterococcus*, and *Oscillospira*, were most closely related to the changed metabolites in the serum of OSR vs. OS group (*P* < 0.05). In particular (**Figures 7E**), bacteria of the *Lactobacillus* genera were negatively correlated with the concentration of alpha-muricholic acid, kynurenine (tryptophan degradation derivatives), 3-phenoxypropinoic acid, and positively correlated with 2-methylbutyroylcarnitine. Bacteria of the *Enterococcus* genera were negatively related to L-cysteinesulfinic acid (oxidative post-translational modification), kynurenine, 3-phenoxypropinoic acid, and positively related to methylbutyroylcarnitine concentration. *Anaerotruncus* genera were mostly related to sotalol, L-cysteinesulfinic acid, kynurenine (tryptophan kynurenine pathway), beta-muricholic acid, 3-Phenoxypropinoic acid, and nucleotide metabolism process (UMP, uridine 5-diphosphate).

### FMT From Resveratrol Diet Ameliorates Egg-Laying Rate of Layers During an OS Challenge

Fecal microbiota derived from oral CON, OS, and OSR-treated layers were transplanted into antibiotics-treated layers. Compared with the FMTOS group, FMTOSR restored the reduction in the egg-laying rate, serum tryptophan levels, and antioxidant enzyme activities (GSH-ST, GSH-PX, and GSH) (**Figures 8A, B, E, F**; *P* < 0.05). Serum concentration of protein and lipid oxidation products (PC and MDA) and kynurenine was lower in the FMTOSR than in FMTOS layers (**Figures 8C, D**; *P* < 0.05).

### Intestinal Microbiota Response to FMT

**Figure 9:**
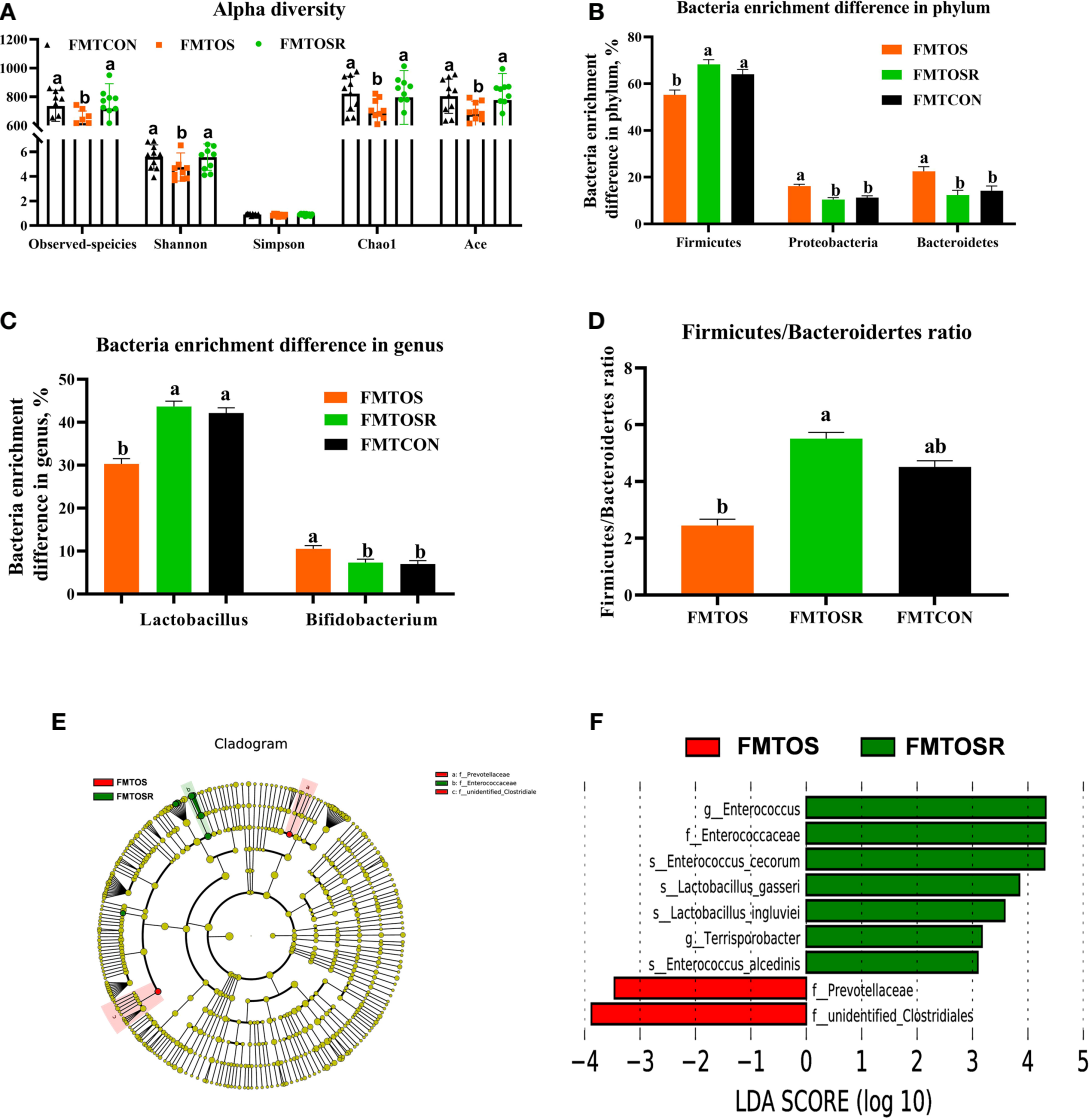
Gut microbiota in response to microbiota transplantation from resveratrol supplementation diet (Exp.2). Data are means ± SEM represented by vertical bars or plot individual values ± SEM. **(A)** Alpha diversity, **(B,C)** Bacteria enrichment difference in phylum and genus, **(D)** Firmicutes/Bacteroidertes ratio, **(E, F)**Linear discrimination analysis coupled with effect size (LEfse) identified most differentially abundant taxa in the cecum with LDA significant threshold > 4 were shown. (Red) FMTCON enrichment taxa; (Green) FMTOS enrichment taxa; (Blue) FMTOSR enrichment taxa. CON = control, OS = oxidative stress (injection of 800 μmol/kg BW of tBHP), OSR = CON + 600 mg/kg resveratrol; FMTCON, FMTOS and FMTOSR indicated the microbiota-depleted layers were received microbiota transplantations from donor layer treated CON diet, tBHP and OSR, individually; Statistical significance was evaluated by the Turkey’s T-test, *p* < 0.05.

Compared with the FMTOS group, FMTOSR increased the Ace, Chao1, and Shannon indexes (**Figure 9A**; *P* < 0.05). As shown by PCoA analysis, cecal microbiota in FMTOS and FMT OSR had an obvious clustering (**Supplementary Figure 3B**; P < 0.05). Compared with FMTOS, at the phylum level, FMTOSR significantly increased relative abundance of Firmiuctes (phylum), *Bacillus* (genus), *Lactobacillu_aviarius* (species), *Lactobacillu_salivarius* (species) but decreased Bacteroidetes (phylum) and Proteobacteria (phylum), *Alistipes* (genus), and *Bacillus_velezensis* (**Figures 9B–F**; **Supplementary Figures S3–A–D**; *P* < 0.05).

## Discussion

Oxidative stress occurs when increased ROS levels disrupt cellular redox circuits, resulting in disturbances of redox-regulated cellular processes and/or oxidatively damage to cellular macromolecules (39, 40). Ovary function plays a central role in fertility, and ovarian function depends on the maintenance and normal development of ovarian follicles (11, 41–43). Accelerated metabolism occurs in rapidly proliferating granulosa cells (GCs) within developing follicles, leading to increased ROS production (1, 44). Accumulating evidence demonstrates that oxidative stress-induced granulosa cell (GCs) apoptosis represents a common reason for follicular atresia by diverse stimuli (such as alcohol, radiation, chemotherapeutic drugs, and smoking) as well as malnutrition and obesity (1, 8, 44, 45). Several studies have suggested that RV can play a role in the improvement of ovarian function by improving the follicular environment resulting in a decreased follicle atresia rate in mice and layers (8, 46). This study was conducted to examine the role of RV in protecting ovarian follicles from atresia *via* the SIRT1-FoxO1/P53 pathway and the involvement of gut microbiota in this process. Oxidation products including PC, MDA, and 8-OHdG have been commonly used to characterize the OS of animals (47, 48). The markedly elevated MDA, PC, and 8-OHdG levels along with significantly lower levels of T-SOD, GSH-PX, GSH-ST, and T-AOC activities within the ovary and serum of tBHP challenged layers (OS group) suggest the occurrence of oxidative damage, whereas RV reversed these changes, indicating that RV may alleviate t-BHP induced oxidative stress in layers. This result is in accordance with our previous study (8), which indicated that intraperitoneal injection of 800 μmol/kg BW tert-butyl hydroperoxide (tBHP) can be used to establish an OS model in layer hens. Also, compared with the CON layer, serum and ovarian proinflammatory cytokines (IL-1β, IL-6, TNF-α, and IFN-γ) were elevated in tBHP-treated layers, thus confirming the positive relationship between OS and the inflammation reaction (49). Aberrant lipid metabolism is suggested to be related to inflammation and OS (50). It was also observed that RV reduced inflammation cytokine secretion induced by tBHP in the current study. Inflammation is an adaptive response, which can be triggered by various signals, such as tissue injury or microorganism invasion. Dietary RV supplementation was able to exert potent anti-inflammatory activity by the suppression of production of proinflammatory cytokines (IL-1α, IL-6, IFN-γ, and TNF-α) *in vitro* and *in vivo* (51–53). Similarly, the RV was also observed to depress proinflammatory cytokine expression in serum (IL-1β and IFN-γ) and ovary (IL-1β, IL-6, IFN-γ, and TNF-α) in the current study. Rencber etal. (54) also found that RV decreased serum and ovary tissue TNF-α concentrations when compared to the DHEA-induced PCOS (polycystic ovary syndrome) rats. The reason why this results in proinflammatory cytokines is not exactly parallel in serum and ovary tissue (especially for TNF-α and IL-6) is not clear. Further study is necessary to research the mechanism underlying this.

In this study, we also observed that dietary RV improved layers’ reproductive performance (as indicated by higher egg-laying rate), hormone secretion levels (higher estradiol, FSH, and IGF-1), and led to a larger ovary index and follicle count compared to layers that underwent the t-BHP oxidative stress challenge. Oxidative stress (induced by tBHP and high levels of molybdenum and vanadium) was also found to decrease egg production in layers and decrease serum concentrations of key reproductive-related hormones (estradiol, FSH, and IGF-1), while the supplementation of antioxidants (tea polyphenols) was able to reverse the adverse effect by improving the antioxidant capacity and gut microbiota balance in our previous studies (7, 8, 55). Studies in mammals have found that oxidative stress can reduce the number of follicles in each stage of the ovarian cycle and impair ovarian function (11, 56, 57). Additionally, previous studies have also demonstrated that oxidative stress led to a reduction in hormone secretion (including lower serum FSH, LH, and higher IGF-1 levels) and impairs the glutathione redox cycle (56). Estradiol and FSH are known to activate antioxidant defense systems scavenging ROS in many organs and systems, especially in the ovary (44, 58). The results of this study indicate that dietary RV can maintain reproductive function by sustaining the secretion of key reproductive hormones in layers during an OS challenge.

SIRT1 is an oxidized nicotinamide adenine dinucleotide (NAD^+^)‐dependent protein deacetylase, and it plays an important role in protection against stress and aging and has been involved in the protective effects of RV (22, 23). In the current study, we observed that melatonin levels were decreased by the OS challenge and that dietary supplementation of RV could alleviate the negative impact of OS and improve ovarian functionality in tBHP challenged layers. SIRT1 is one of the main modulators of metabolism and resistance to oxidative stress (21–23). As a promoter of SIRT1, RV upregulates SIRT1 expression in the ovaries, which is associated with protection against OS (59–63). Our results also suggest that dietary RV can upregulate the Nrf2-ARE signaling pathway, which is in agreement with previous studies (27, 64).

Additionally, in this study, we observed that the tBHP challenge decreased tryptophan and melatonin levels and increased kynurenine levels in serum. In a previous study, we also observed that the tBHP challenge disrupted tryptophan metabolism (8). Tryptophan is highly susceptible to oxidative degradation, and its metabolism is involved in the regulation of intestinal homeostasis, immunity, and neuronal function (14). There are two main metabolic pathways for tryptophan: the 5-HT pathway, which is used for the synthesis of 5-hydroxytryptophan (5-HT) and melatonin (65); and the kynurenine pathway, which accounts for 90% of tryptophan catabolism. The kynurenic pathway is rate-limited by its first enzyme, Trp 2,3-dioxygenase (TDO) in the liver and IDO elsewhere; the IDO is regulated by IFN-γ, other cytokines, and by nitric oxide (14). Chronic stress and infections can divert the available tryptophan toward the kynurenic pathway by increasing the IDO enzyme activity and thereby lowering 5-HT and melatonin synthesis (31). As observed in the current study, IDO1 in the jejunum was enhanced by the OS challenge and reversed by the dietary supplementation of RV, which may suggest that the tryptophan-kynurenine pathway was also involved in this process. Tryptophan is also the precursor of melatonin, which can be synthesized in the intestine (66, 67). Therefore, the decreasing levels of tryptophan in serum may be the result of the higher Mel in serum. The intestinal microbiota plays a critical link between the health of the host and environmental cues. Previous studies suggested that the intestinal microbiota is sensitive to stress and aging, which can dramatically shape or reverse intestinal microbiota profiles in human and animal models (47, 68, 69). In the present study, we observed that dietary RV or transplant digesta from RV-supplemented birds enhanced the bacteria α-diversity of layers under OS, and improved the enrichment of Firmicutes (phylum), *Lactobaccillus* (genus), and *Bifidobacterium* (genus). Previous studies have also demonstrated that feeding mice with RV during a colitis challenge could increase the relative abundance of *Lactobacillus*, Bifidobacterium, as well as Clostridium, and promote the metabolism of dietary fiber into SCFAs (70–72). As observed in our study, the bacteria genera, including *Lactobacillus*, *Harryflintia*, *Enterococcus*, and *Anearotruncus* were highly correlated to the changes in serum metabolites observed in the OSR and OS groups. In particular, bacteria of the *Lactobacillus* genera were negatively correlated with the concentration of alpha-muricholic acid and kynurenine. As shown in previous studies, a significant portion (about 90%) of ingested resveratrol reaches the colon in its intact form and is subsequently subjected to gut fermentation (73). Among several possible tryptophan metabolism routes, the kynurenine pathway can be influenced by the gut microbiota. An imbalance in gut bacterial population composition (dysbiosis) or changes in the production of melatonin (circadian disruption) alters estrogen levels. Several bacteria belonging to *Lactococcus*, *Lactobacillus*, *Streptococcus*, and *Baccillus* have been reported to be able to produce serotonin and melatonin by expressing tryptophan synthetase (29, 74). On the other hand, it has been shown that these changes in intestinal microbiota can stimulate the kynurenine pathway by moving tryptophan away from the melatonergic pathway, thereby reducing circulating melatonin levels. Microbial metabolism produces melatonin directly and, on the other hand, gut bacteria indirectly produce short-chain fatty acids (SCFAs) that stimulate the production of serotonin, which is then converted into melatonin. In the current study, dietary RV during an OS challenge also increased the SCFA levels, which may also be attributed to the higher levels of melatonin levels in serum. To better explore the relationship between microbiota and RV, microbiota from CON, OS, and OSR were transplanted into layers depleted with intestinal microbiota by antibiotics. Data revealed similar effects on colonic microbial composition, SCFA concentration, oxidative status, and serum tryptophan levels in layers from CON, OS, and OS+RV groups. Thus, our findings indicate a causal role between gut microbiota on ovarian function during an oxidative stress challenge.

## Conclusion

**Figure 10:**
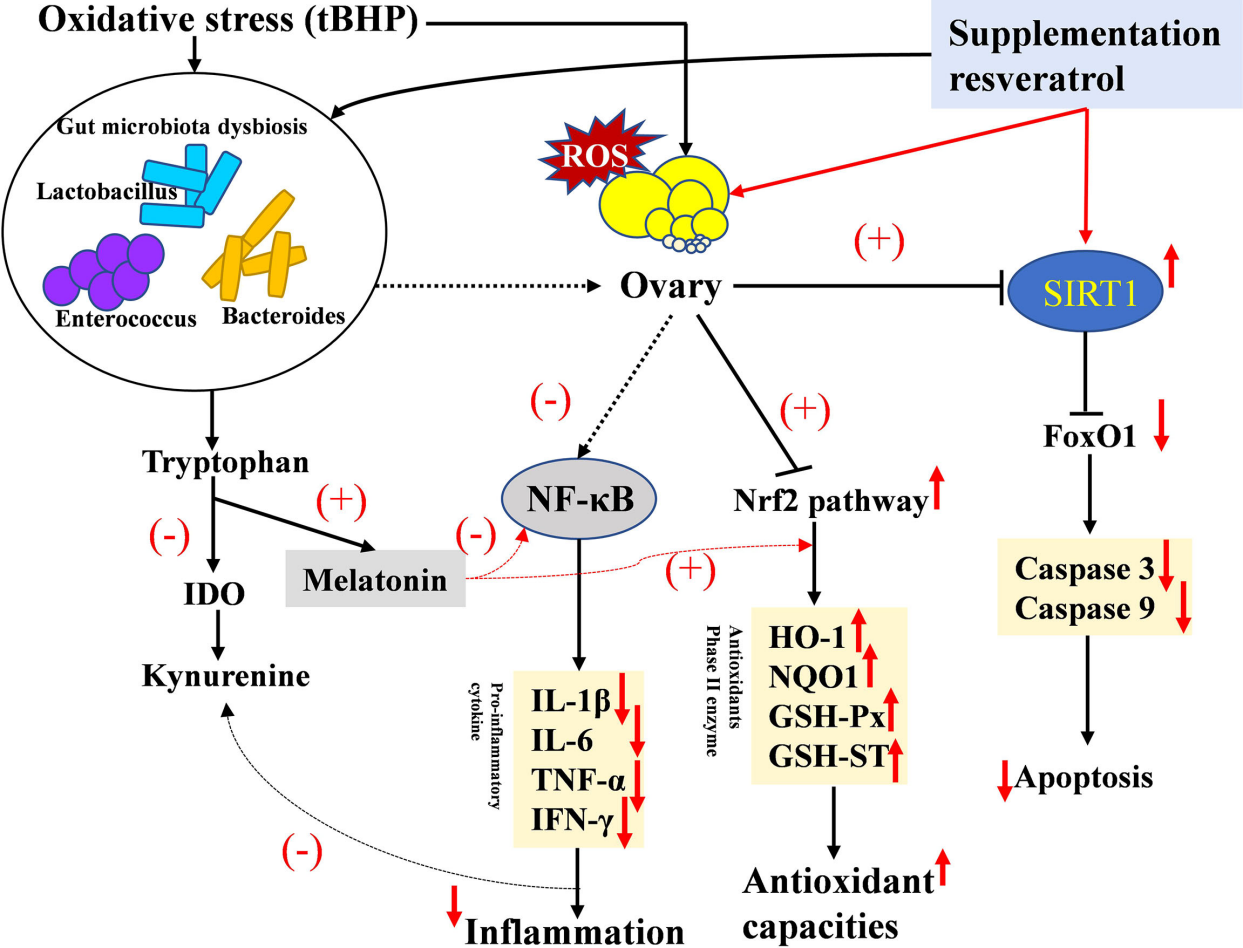
Graphical abstract of this article. Proposed mechanism of oxidative stress in lowering ovary function and the ameliorating effect of melatonin in the layer model. The up arrow (↑,+) and down arrow (↓,-) represent upregulation and downregulation effects of oxidative stress and resveratrol, respectively.

In conclusion, we found that OS could decrease fertility and induce gut microbiota dysbiosis and inflammation by disrupting the tryptophan-kynurenine pathway in a layer model. We also identified a novel mechanism linking intestinal microbiota to dietary RV and improved oxidative status and ovarian function. RV was able to reverse the impact of OS through the SIRT1-P53/FoxO1 and Nrf2-ARE pathway (**Figure 10**). The microbiota and its related tryptophan-kynurenine pathway were involved in mediating the observed mitigating effect of dietary RV during an OS challenge.

## Data Availability Statement

The original contributions presented in the study are publicly available. This data can be found here: http://www.ncbi.nlm.nih.gov/bioproject/839197.

## Ethics Statement

The animal study was reviewed and approved by Animal Care and Use Committee of Sichuan Agricultural University (SICAU-2020-041). Written informed consent was obtained from the owners for the participation of their animals in this study.

## Author Contributions

JW and RJ conceived and designed the experiments; JW, RJ and YZ performed the experiments; JW, RJ and SB analyzed the data and wrote the paper; SB, ZQ, XM, SX, HY, LL and KZ helped revise this manuscript. All authors read and approved the final manuscript.

## Funding

This work was financially supported by the National Natural Science Foundation of China (31872792), and Sichuan Science and Technology Program (2022YFH0070).

## Conflict of Interest

The authors declare that the research was conducted in the absence of any commercial or financial relationships that could be construed as a potential conflict of interest.

## Publisher’s Note

All claims expressed in this article are solely those of the authors and do not necessarily represent those of their affiliated organizations, or those of the publisher, the editors and the reviewers. Any product that may be evaluated in this article, or claim that may be made by its manufacturer, is not guaranteed or endorsed by the publisher.

## Abbreviations

AF, atretic follicle; Bcl-2, B-cell lymphoma-2; CON, control; FoxO1, forkhead box O1; GSH, glutathione; GSH-ST, glutathione s-transferase; HO-1, heme oxygenase 1; IDO1, indoleamine 2,3-dioxygenase (IDO) 1; IL, interleukin; MDA, malondialdehyde; NF-κB, nuclear factor κ; NQO-1, NAD(P)H:quinine oxidoreductase 1; Nrf2, nuclear factor E2-related factor 2; 8-OHdG, 8-hydroxy-2′-deoxyguanosine; OS, oxidative stress; PC, protein carbonyl; PF, primary follicle; PHF, prehierarchical follicle; PMF, primordial follicle; ROS, reactive oxygen species; RV, resveratrol; SIRT1, silent information regulator 1; SOD, superoxide dismutase; T-AOC, total antioxidant capacity; tBHP, tert-butyl hydroperoxide; TNF-α, tumor necrosis factor-α.
