# Supplementary figures and images for: Resveratrol Alleviating the Ovarian Function Under Oxidative Stress by Alternating Microbiota Related Tryptophan-Kynurenine Pathway
Source: Front Immunol. 2022 Jul 13;13:911381. doi: 10.3389/fimmu.2022.911381 (PMC9327787; doi:10.3389/fimmu.2022.911381)

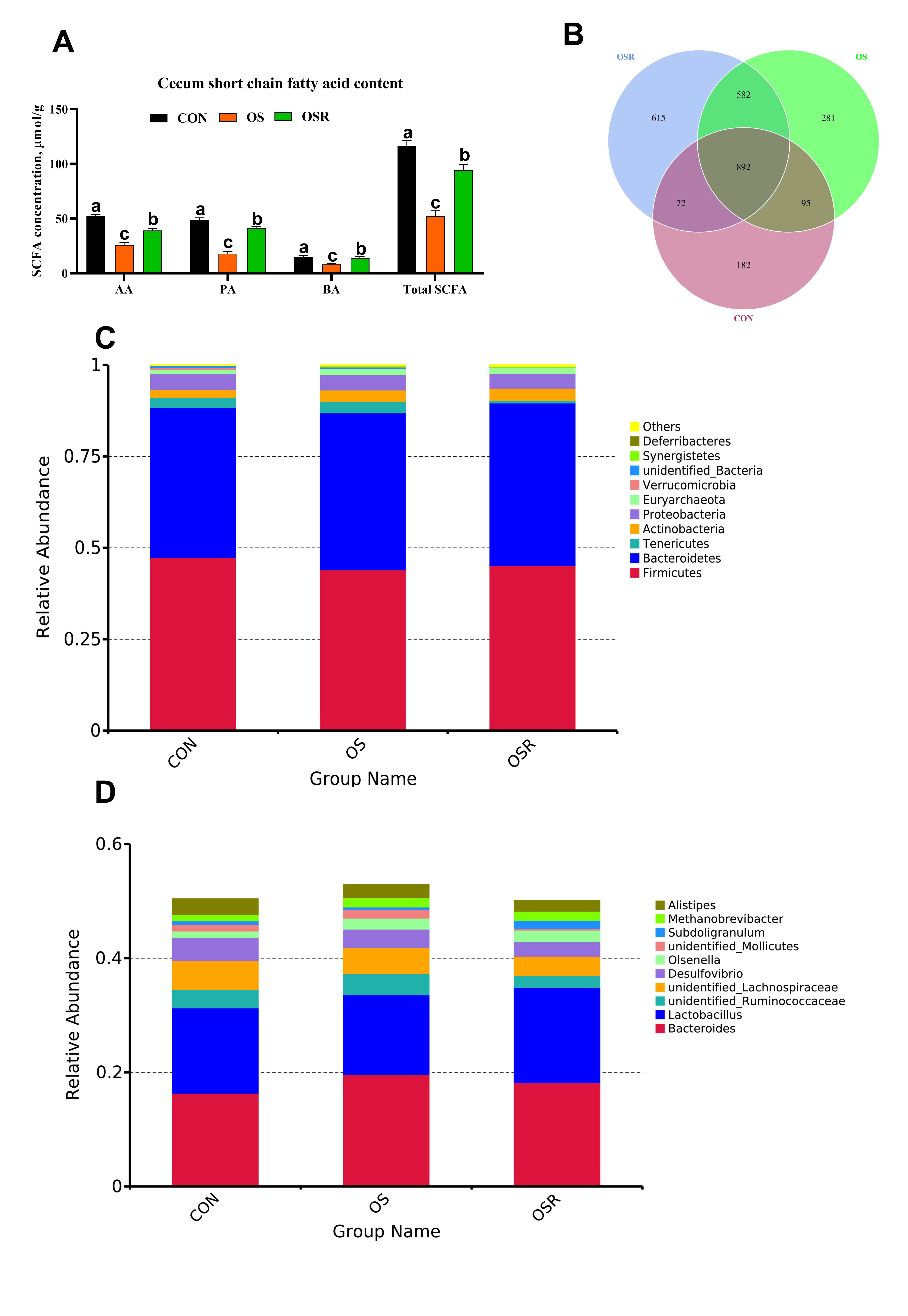

Supplement: Supplementary file 1 [file Image_1.jpg]

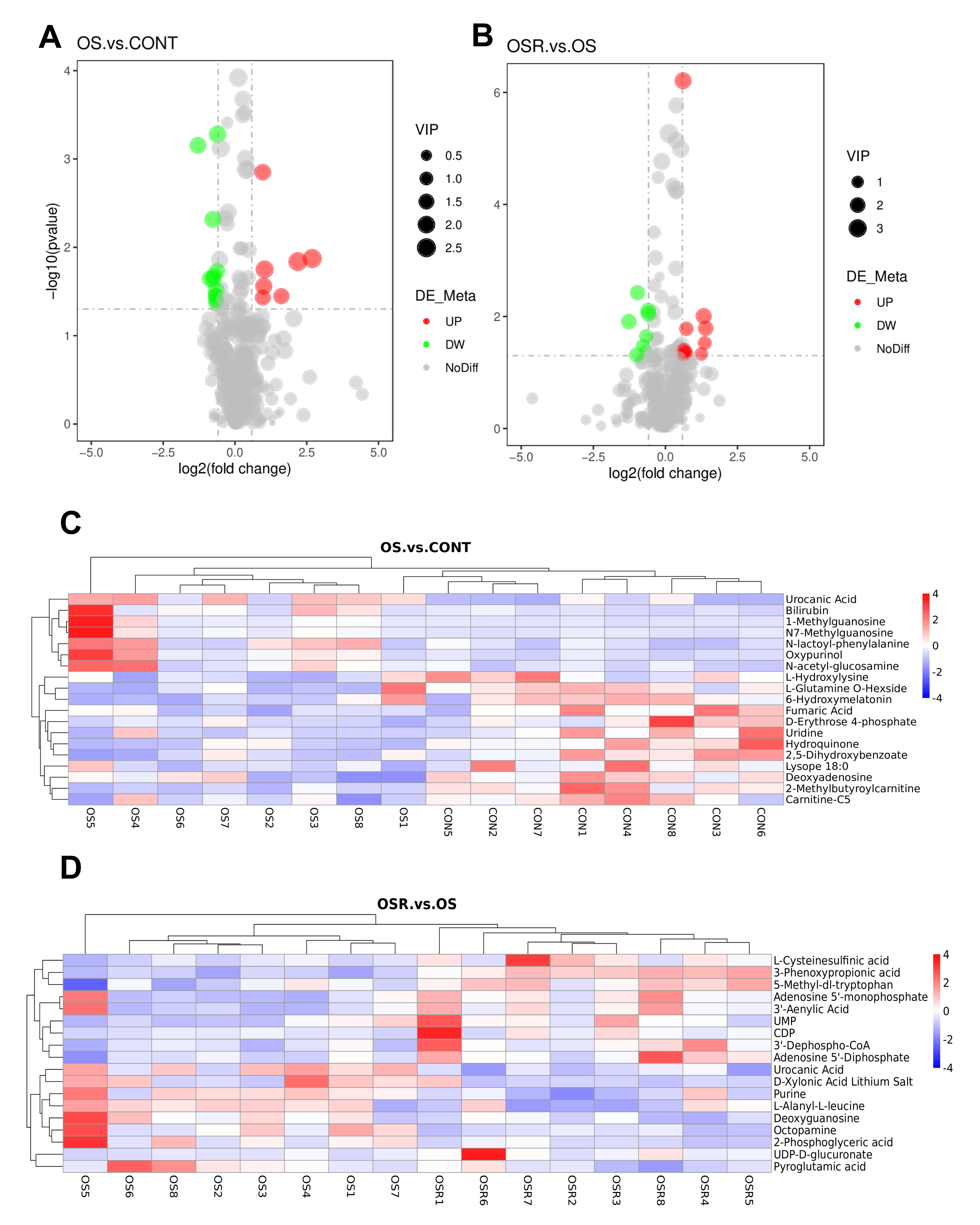

Supplement: Supplementary file 2 [file Image_2.jpg]

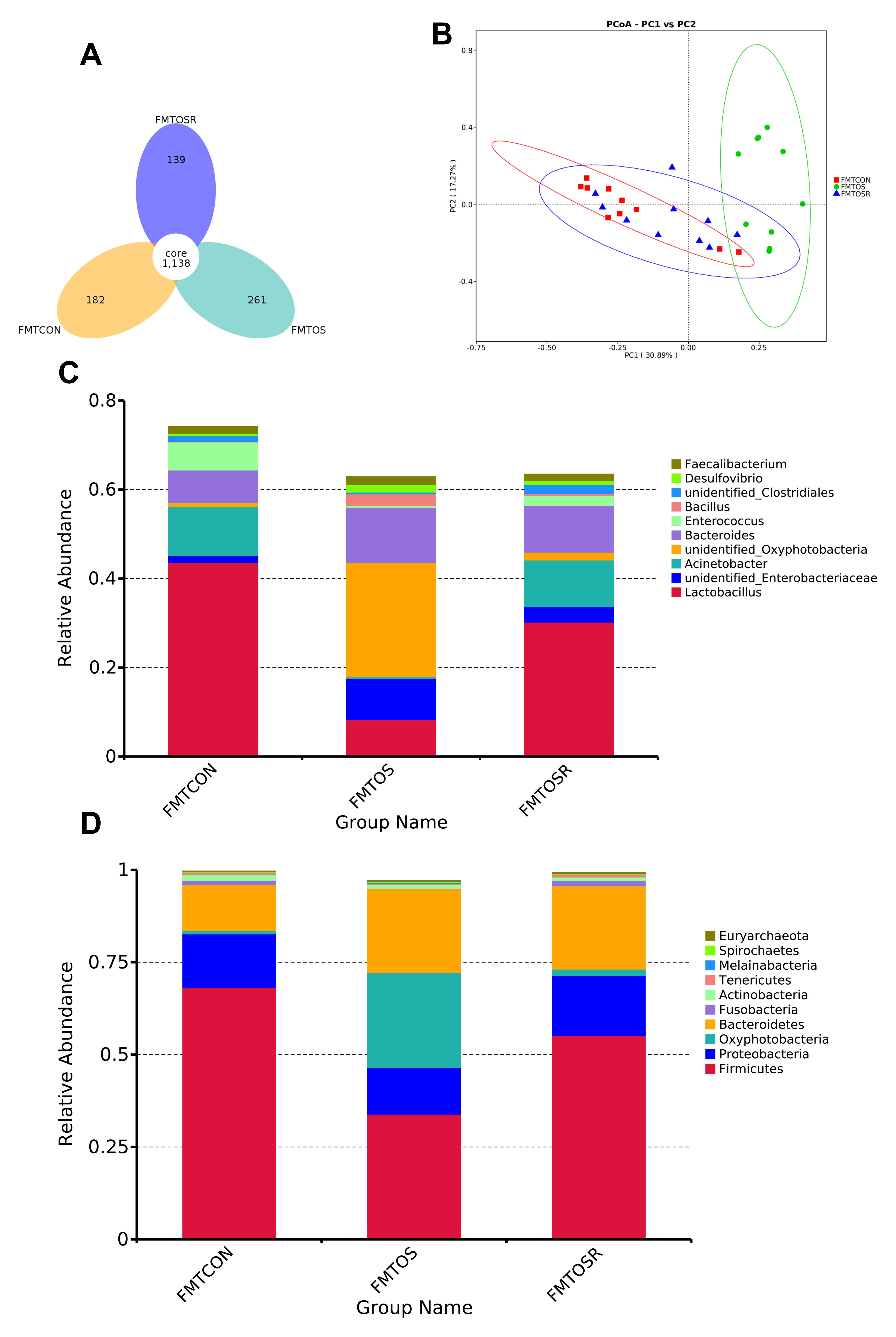

Supplement: Supplementary file 3 [file Image_3.jpg]
