# Supplementary material for: Resveratrol Alleviating the Ovarian Function Under Oxidative Stress by Alternating Microbiota Related Tryptophan-Kynurenine Pathway
Source: Front Immunol. 2022 Jul 13;13:911381. doi: 10.3389/fimmu.2022.911381 (PMC9327787; doi:10.3389/fimmu.2022.911381)
Supplement: Supplementary file 4 [file DataSheet_1.docx]

| **Table S1. Composition and nutrient levels of basal diet** | |
| --- | --- |
| Item, % | Amount |
| Corn | 57.72 |
| Wheat bran | 5.00 |
| Soybean oil | 2.00 |
| Soybean meal | 23.30 |
| CaCO_3_ | 9.70 |
| Calcium hydrophosphate | 1.05 |
| NaCl | 0.40 |
| L-Lysine·HCl | 0.10 |
| DL-Methionine | 0.10 |
| Tryptophan | 0.05 |
| Threonine | 0.05 |
| Choline chloride | 0.10 |
| Vitamin premix^1^ | 0.03 |
| Mineral premix^2^ | 0.40 |
| Total | 100.00 |
| Analyzed nutrient levels, % |  |
| ME, kcal/kg | 2650.00 |
| Crude protein | 15.50 |
| Calcium | 4.00 |
| Total phosphorus | 0.53 |
| Lysine | 0.77 |
| Methionine | 0.36 |
| Methionine + cysteine | 0.58 |
| ^1^Provided per kilogram of diet: vitamin A, 8000 IU; vitamin B_1_, 0.4 mg; vitamin B_2_, 1.2 mg; D-pantothenate, 5 mg; vitamin B_6_, 7.55 mg; vitamin B_12_, 6 μg; vitamin D_3_ 2000 IU; vitamin E 5 IU, vitamin K_3_ 1 mg; biotin, 4 mg; niacin acid 7 mg; folic acid 100 μg. | |
| ^2^Provided per kilogram of diets: Cu (as copper sulfate)：8 mg, Fe (as ferrous sulfate) 60 mg，Mn (as manganese sulfate) 60 mg，Zn (as zinc sulfate) 80 mg，I (as potassium iodide) 1 mg，Se (as sodium selenite) 0.30 mg. | |
| ^3^Calculated according to NRC (1994). | |

| **Table S2.** Related gene and primer information | | | | |
| --- | --- | --- | --- | --- |
| Genes^1^ | Orientation | Primer Sequences (5'-3') | Product size | Accession number^2^ |
| *Caspase 3* | Forward | AAAGATGGACCACGCTCAGG | 204 | NM_204725 |
|  | Reverse | TGAACGAGATGACAGTCCGG |  |  |
| *Caspase 9* | Forward | TATGGTGGAGGACATGCAGA | 99 | XM_424580.5 |
|  | Reverse | AATATTGGGAAGGCCTGCTT |  |  |
| *Bcl-2* | Forward | ACCATGAATGAAACCGTGCC | 181 | NM_205339.2 |
|  | Reverse | TTGTCGTAGCCTCTTCTCCC |  |  |
| *Bax* | Forward | GTACGTCAATGTGGTCACCC | 210 | XM_015274882 |
|  | Reverse | TGGGATAATGCTGGGGTTGA |  |  |
| *SIRT1* | Forward | TAGCCAATGGTTTCCACTCC | 149 | NM_001004767.1 |
|  | Reverse | AAGAATTGTCCGTGGGTCTG |  |  |
| *FoxO1* | Forward | AAGAGCGTGCCCTACTTCAA | 125 | NM_204328.1 |
|  | Reverse | TTCCCTGTTCCCTCATTCTG |  |  |
| *P53* | Forward | TACTCCCCGGTGCTGAATAA | 134 | NM_205264.1 |
|  | Reverse | TAGCCAATGGTTTCCACTCC |  |  |
| *Nrf2* | Forward | GTACGTCAATGTGGTCACCC | 143 | NM_205117.1 |
|  | Reverse | TGTGTGTGATTCAACCCGACT |  |  |
| *HO-1* | Forward | TTAATGGAAGCCGCACCACT | 210 | NM_205344.1 |
|  | Reverse | TTGGCAAGAAGCATCCAGA |  |  |
| *NQO-1* | Forward | TCCATCTCAAGGGCATTCA | 210 | NM_001277619.1 |
|  | Reverse | GTTCAATGCCGTGCTCTCAC |  |  |
| *Keap1* | Forward | TTCCACGCCTGCATCAACT | 135 | XM_010728179 |
|  | Reverse | GGTTTGTGCAGGGTGAGG |  |  |
| *β-actin* | Forward | ATCCGGACCCTCCATTGTC | 152 | NM_205518.1 |
|  | Reverse | AGCCATGCCAATCTCGTCTT |  |  |
| ^1^Abbreviation indicated that: Nrf2 = nuclear factor erythroid-2 related factor 2, NQO1 = NAD(P)H quinone dehydrogenase 1, HO-1 = Heme oxygenase-1, Bcl-2 = B-cell lymphoma-2, β-actin=beta-actin (reference gene). | | | | |
| ^2^GenBank accession number for sequence from which primers were designed. | | | | |

| **Table S3.** Serum different metabolites in OS group compared to controls | | | | | |
| --- | --- | --- | --- | --- | --- |
| Name | FC | P-value | ROC | VIP | Up_Down |
| Hydroquinone | 0.41 | <0.01 | 0.91 | 1.84 | down |
| L-Hydroxylysine | 0.55 | 0.02 | 0.81 | 1.67 | down |
| L-Glutamine O-Hexside | 0.59 | 0.02 | 0.84 | 1.69 | down |
| 2,5-Dihydroxybenzoate | 0.59 | <0.01 | 0.89 | 1.92 | down |
| D-Erythrose 4-phosphate | 0.60 | 0.02 | 0.86 | 1.62 | down |
| Carnitine-C5 | 0.62 | 0.04 | 0.81 | 1.37 | down |
| Lysope 18:0 | 0.63 | 0.04 | 0.84 | 1.35 | down |
| 6-Hydroxymelatonin | 0.63 | 0.04 | 0.78 | 1.46 | down |
| Deoxyadenosine | 0.64 | 0.03 | 0.81 | 1.73 | down |
| Uridine | 0.65 | 0.02 | 0.88 | 1.68 | down |
| Fumaric Acid | 0.65 | 0.04 | 0.77 | 1.79 | down |
| 2-Methylbutyroylcarnitine | 0.66 | <0.01 | 0.98 | 2.05 | down |
| Kynurenine | 6.50 | <0.01 | 0.97 | 2.17 | up |
| 1-Methylguanosine | 6.45 | 0.01 | 0.98 | 2.52 | up |
| N7-Methylguanosine | 4.56 | 0.01 | 0.95 | 2.48 | up |
| Bilirubin | 3.07 | 0.04 | 0.78 | 1.61 | up |
| Oxypurinol | 2.05 | 0.02 | 0.89 | 2.20 | up |
| Urocanic Acid | 1.96 | <0.01 | 0.94 | 1.78 | up |
| N-lactoyl-phenylalanine | 1.96 | 0.04 | 0.77 | 1.55 | up |

| **Table S4.** Serum different metabolites in OSR group compared to OS controls | | | | | |
| --- | --- | --- | --- | --- | --- |
| Name | FC | P-value | ROC | VIP | Up_Down |
| 2-Phosphoglyceric acid | 0.42 | 0.01 | 0.91 | 2.10 | down |
| Kynurenine | 0.42 | <0.01 | 0.92 | 2.07 | down |
| L-Alanyl-L-leucine | 0.50 | 0.05 | 0.81 | 1.85 | down |
| Urocanic Acid | 0.51 | 0.01 | 0.88 | 1.90 | down |
| D-xylonic acid lithium salt | 0.58 | 0.03 | 0.77 | 1.34 | down |
| Deoxyguanosine | 0.63 | 0.02 | 0.80 | 1.61 | down |
| Pyroglutamic acid | 0.63 | 0.04 | 0.77 | 1.25 | down |
| Purine | 0.66 | 0.01 | 0.86 | 2.06 | down |
| Octopamine | 0.67 | 0.01 | 0.89 | 2.08 | down |
| 3'-Dephospho-CoA | 2.63 | 0.02 | 0.91 | 1.98 | up |
| UDP-D-glucuronate | 2.58 | 0.03 | 0.84 | 1.47 | up |
| L-Cysteinesulfinic acid | 2.51 | 0.01 | 0.88 | 2.23 | up |
| Cytidine 5'-(trihydrogen diphosphate) | 2.37 | 0.05 | 0.84 | 1.37 | up |
| Adenosine 5'-Diphosphate | 1.66 | 0.02 | 0.88 | 1.64 | up |
| Uridine 5'-monophosphate | 1.63 | 0.04 | 0.78 | 1.57 | up |
| 5-Methyl-dl-tryptophan | 1.57 | 0.04 | 0.91 | 1.91 | up |
| Adenosine 5'-monophosphate | 1.55 | 0.04 | 0.80 | 1.52 | up |
| 3-Phenoxypropionic acid | 1.53 | 0.01 | 1.00 | 2.55 | up |
| 3'-Aenylic Acid | 1.53 | 0.05 | 0.81 | 1.47 | up |
